# Response to Letter Regarding Article, "Brugada-type Electrocardiographic Pattern Induced by Electrocution"

**Published:** 2009-03-15

**Authors:** R Rangaraj, Nagaraja Moorthy, Shivanand S Patil, C N Manjunath

**Affiliations:** Department of cardiology, Sri Jayadeva Institute of Cardiology, Bangalore, India

**Keywords:** Electrocution, Brugada syndrome, pericarditis

Pericarditis as a differential diagnosis of the "Brugada-type Electrocardiographic Pattern Induced by Electrocution" reported by us  [1] raised by Shomu Bohora needs appreciation. The electrocardiogram in isolation favours the diagnosis of pericarditis. However the clinical background, lack of symptoms or clinical signs and absence of evolution of ECG features through stages do not favour pericarditis.

However the early repolarisation pattern and non specific ST-T changes as a part of transient electrical injury of myocardium would be a better explanation for the ECG changes noted. The ST-T changes in right sided chest leads obviously simulates Brugada type pattern.

The electrocution can result in non-specific electrocardiographic changes and varied ST-T changes mimicking pericarditis, early repolarisation, Brugada type pattern, myocardial infarction etc. However these changes needs to be correlated with the clinical background before the final diagnosis is made since majority of these changes are due to electrical injury of heart.
